# The Influence of Crown Ether and Alcohol on Unsaturation and Molar Mass of Poly(propylene oxide)s Prepared by Use of Potassium* t*-Butoxide: Reinvestigation of Chain Transfer Reactions

**DOI:** 10.1155/2016/3727062

**Published:** 2016-07-26

**Authors:** Zbigniew Grobelny, Andrzej Swinarew, Justyna Jurek-Suliga, Kinga Skrzeczyna, Jadwiga Gabor, Marta Łężniak

**Affiliations:** ^1^Institute of Chemistry, Faculty of Mathematics, Physics and Chemistry, University of Silesia in Katowice, 40-007 Katowice, Poland; ^2^Institute of Material Science, Faculty of Computer Science and Material Science, University of Silesia in Katowice, 40-007 Katowice, Poland

## Abstract

Potassium* t*-butoxide dissolved in tetrahydrofuran effectively initiates homogeneous polymerization of propylene oxide at room temperature. Unsaturation and molar mass (*M*_*n*_) of the polymers prepared depend on the presence of additives, such as macrocyclic ligand 18-crown-6 (L) and* t*-butanol. Application of the ligand alone results in distinct increase of unsaturation and decrease of *M*_*n*_, whereas use of* t*-BuOH leads to simultaneous decrease of unsaturation and *M*_*n*_. Activation of* t*-BuOK/*t*-BuOH system with the ligand causes further decrease of unsaturation, that is, from 12.0 to 3.5 mol % for OK/OH (1/3) and OK/OH/L (1/3/2) systems, respectively. Unexpectedly, *M*_*n*_ of the polymers obtained does not practically change (~4800). This result differs from that reported earlier for neat PO polymerization initiated potassium 1-methoxy-2-propoxide/1-methoxy-2-propanol, in which in the presence of the same ligand *M*_*n*_ increases to ~12 400 for the same ratio of reagents. The mechanism of studied processes was discussed.

## 1. Introduction

Polymers of propylene oxide (PPOs) are important polyethers, which have several applications, for example, as impact modifiers, dispersant agents, deemulsifiers, fuel additives, wetting agents, lubricants, adhesives, and biomedical applications [1–5]. PPOs are produced mainly by anionic polymerization of PO with use of potassium alkoxides in bulk or in solution. Initiators with two active sites, that is, KOH/glycol system or dipotassium glycoxides, are particularly interesting because they are used in the synthesis of triblock copolymers with wide applications as surfactants [6] or in synthesis of macromonomers for fabrication of polyurethane elastomers [7]. Use of KOH/glycerol system allows preparation of polyether-triols for synthesis of crosslinked polyurethane foams [8].

**Scheme 1:**
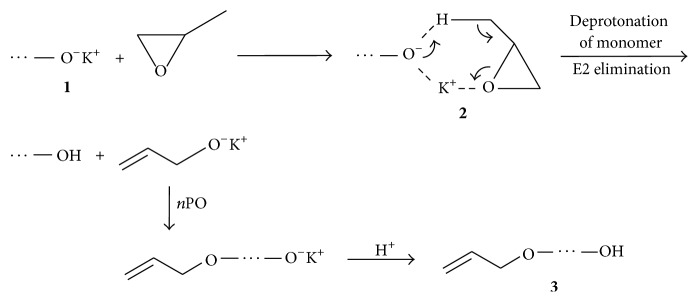
Mechanism of the chain transfer reaction to the monomer.

A disadvantage of anionic polymerization of PO is side reaction, that is, chain transfer to the monomer, which markedly decreases molar mass of polymers (*M*_*n*_ = 2000–3000) and produces unsaturated polyether polyols, so-called “monols” (Scheme 1) [7, 8].

The general properties of polyurethanes can be greatly improved by the use of polyether polyols with reduced monol content. It is possible by lowering the temperature and initiator concentration or by using suitable solvents [6–8]. More selective catalyst systems which can produce a smaller proportion of monofunctional products are therefore of increasing importance.

**Scheme 2:**
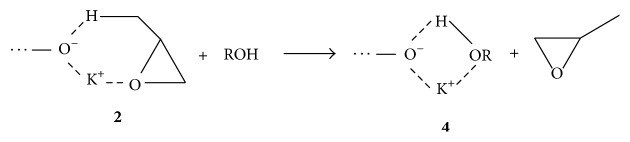
Decomposition of the alkoxide active centre/PO complex** 2** under influence of alcohol.

One of the methods of unsaturation reduction is using for initiation of polymerization mixtures of potassium alkoxides and alcohols [9]. Unsaturation of the polymers markedly decreases due to elimination of PO from complex** 2** because OH groups are stronger ligands for K^+^ than PO (Scheme 2) [8].

Addition of macrocyclic ligand 18-crown-6 (18C6) to the system containing alcohol causes further depression of chain transfer to the monomer resulting in decrease of unsaturation [10]. The polyethers obtained have two to four times lower unsaturation than the polymers synthesized by usual anionic PO polymerization with uncomplexed potassium cations. The minimum unsaturation of the polyether-triols was found in the polymerization initiated with monopotassium salt of glycerin while using 18C6 or cryptand C222 which appeared to be better ligands than dibenzo-18C6 or poly(ethylene glycols) [11]. Moreover, using for initiation a mixture of 1-methoxy-2-propanol and its potassium salt together with 18C6 (1/3/1.94 ratio) PPO with low unsaturation and high *M*_*n*_ = 12 400 was obtained [12].

**Scheme 3:**
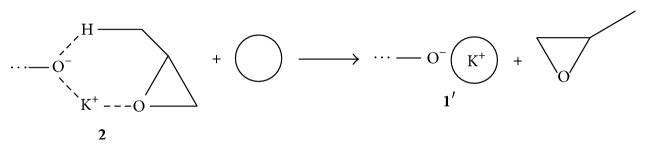
Decomposition of alkoxide active centre/PO complex** 2** in the presence of macrocyclic ligand (a circle denotes ligand).

An explanation of this effect given by Ionescu [8] is the acceleration of propagation and the deceleration of the chain transfer to the monomer. The author proposed that, using a strong complexing agent for K^+^, PO being a soft ligand is eliminated from the complex and thus chain transfer is inhibited (Scheme 3). However, the role of the alcohol in this process was not taken into account.

The aim of the present work was to determine the influence of ligand 18C6 on unsaturation and molar mass of polymers obtained in PO polymerization initiated with potassium* t*-butoxide and, comparatively, with potassium* t*-butoxide/*t*-butanol system. Polymerizations were carried out in mild homogeneous conditions, that is, in tetrahydrofuran solution and room temperature. Mechanism of the reactions was discussed.

## 2. Experimental Part

### 2.1. Materials

Propylene oxide (Aldrich) was dried over CaH_2_ and finally distilled at 307 K (34°C). Anhydrous tetrahydrofuran (THF) (Across Organics) was kept over CaH_2_ and distilled at 339 K (66°C). Potassium* t*-butoxide (1.0 mol/dm^3^ solution in tetrahydrofuran) (Aldrich), coronand 18C6 (1,4,7,10,13,16-hexaoxacyclooctadecane) (Merck),* t-*butanol, and methyl iodide were used for initiation without purification.

### 2.2. Propylene Oxide Polymerization

All syntheses were performed at room temperature in a 50 cm^3^ reactor equipped with a magnetic stirrer and a teflon valve enabling substrates delivery and sampling under argon atmosphere. In the first series of polymerizations the initial concentration of the monomer and initiator was equal to 2.0 and 0.1 mol/dm^3^ but in the second one 10.0 and 0.05 mol/dm^3^, respectively. For example, tetrahydrofuran (15.2 cm^3^) and* t*-BuOK solution (2.0 cm^3^) were introduced into reactor and mixed during 5 min. That solution was used as the initiator when propylene oxide (2.8 cm^3^; 2.3 g; 40 mmol) was added into the reactor. The homogeneous reaction mixture was then stirred several days. After almost complete conversion of the monomer methyl iodide was added to transform alkoxide active centres into the methoxy end groups. After separation of potassium iodide precipitate from the reaction mixture by filtration, THF was evaporated in vacuum from the solution at room temperature yielding a viscous liquid polymer. In an additional experiment the polymerization was carried out with the addition of 18C6 (0.53 g; 2.0 mmol) or* t*-butanol (0.19 cm^3^; 2.0 mmol). The concentration of monomer during the polymerizations was monitored by the 1,4-dioxane method [13]. The final conversion was equal to 99%. The yields of the reactions were 95−98%.

### 2.3. Measurements

100 MHz ^13^C NMR spectra were recorded in CDCl_3_ at 25°C on a Bruker Avance 400 pulsed spectrometer equipped with 5 mm broad-band probe and applying Waltz 16 decoupling sequence. Chemical shifts were referenced to tetramethylsilane serving as an internal standard. To obtain a good spectrum of the polymer main chain exhibiting its microstructural details about 3000 scans were sufficient but in order to observe the signals of the polymer chain terminal groups more than 10 000 scans were necessary. Molar masses and dispersities of polymers were obtained by means of size exclusion chromatography (SEC) on a Shimadzu Prominence UFLC instrument at 40°C on a Shodex 300 mm × 8 mm OHpac column using THF as a solvent. Poly(propylene glycol)s were used as calibration standards.

## 3. Results and Discussion

The results obtained were collected in Table 1. Polymerizations were performed at various initial concentrations of monomer and initiator, that is, 2.0 and 0.1 mol/dm^3^, respectively (1,2,5,6,9–11), as well as 10.0 and 0.05 mol/dm^3^, respectively (3,4,7,8,12–16).

**Figure 1:**
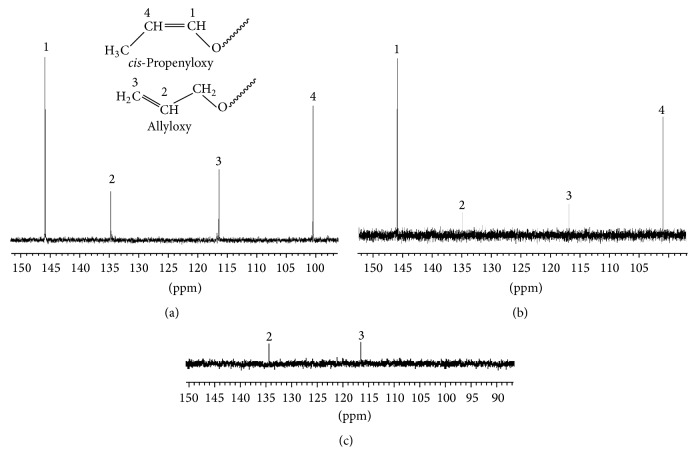
Unsaturated region in ^13^C NMR spectra of polymers: (a) sample 1; (b) sample 2; and (c) sample 9.

Unsaturation of polymers obtained changes in wide range, from 0.2 to 40.1 mol % depending on the presence of additives. Unsaturation is represented by allyloxy starting groups; however, in some polymers also isomeric* cis*-propenyloxy groups were detected and estimated by ^13^C NMR spectroscopy, that is, 5.8 and 25.5 mol % in samples 1 and 2, respectively.  Figure 1 presents ^13^C NMR spectra of mentioned polymers 1 and 2 and, additionally, polymer 9, without* cis*-propenyloxy starting groups.

Activation of* t-*BuOK with the ligand causes distinct increase of polymer unsaturation (samples 2 and 4 in* Set A*). Evidently, in these cases the acceleration of chain transfer to the monomer (which depends on basicity of anions) occurs simultaneously with the acceleration of propagation (which depends on nucleophilicity of anions). However, the acceleration of the first reaction is greater than the acceleration of the second one.

**Scheme 4:**
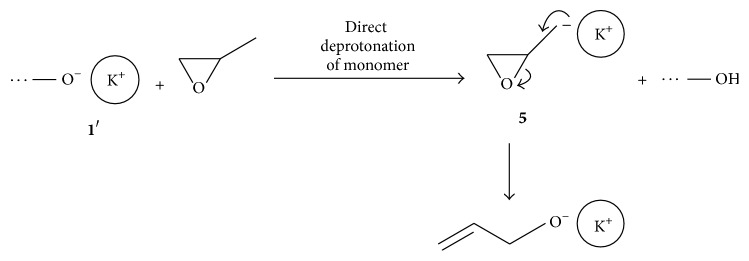
Mechanism of direct deprotonation of the monomer by ligand activated ion pairs.

We assumed that deprotonation of the monomer by ligand activated ion pairs 1′ present in polymer chains or initiator molecules cannot occur by elimination reaction (E2) due to steric hindrance and electronic reasons. However, chain transfer reaction is not inhibited but can be realized successfully by direct deprotonation of the monomer, resulting in distinct increase of polymer unsaturation (Scheme 4). In this reaction extremely reactive potassium glycidoxide** 5** as intermediate is formed, which decomposes by the ring opening in the *α*-position giving potassium allyloxide.

It is worth noting that increase of polymer unsaturation in the presence of ligand is accompanied with decrease of molar masses, due to increase of number of polymer chains caused by chain transfer to monomer. Similar effects were observed earlier by us in the polymerization of PO initiated anhydrous potassium hydroxide [14].

**Scheme 5:**
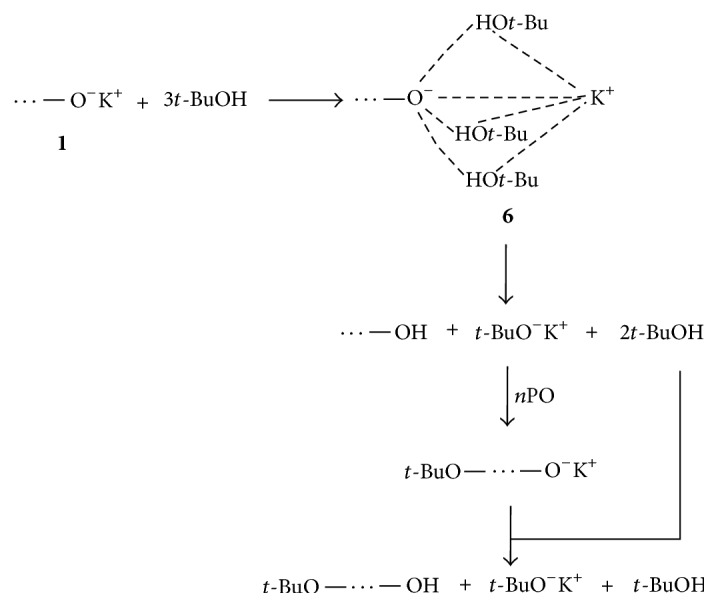
Formation of hydrogen-bonded ion pair with three alcohol molecules** 6** leading to chain transfer to alcohol.

Application of* t*-BuOK*/t*-BuOH mixtures as initiators results in distinct decreasing of polymer unsaturation and *M*_*n*_, especially for 1/3 system (samples 6 and 8 in* Set B*). The course of the process was shown in Scheme 5.

Diminishing of PPO unsaturation was reported previously for PO polymerization initiated monopotassium salt of glycerol mixtures with glycerol at high concentration of the last one [9]. However, results concerning the influence of OH groups on *M*_*n*_ of polymers were not presented in cited work.

**Scheme 6:**
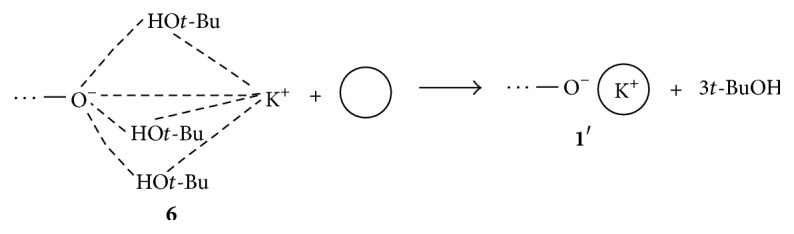
Decomposition of hydrogen-bonded ion pair** 6** in the presence of macrocyclic ligand.

Data concerning* Set C* result from the use of* t*-BuOK*/t*-BuOH (1/3) initiating system containing ligand at K^+^/18C6 = 1/1, 1/2, or 1/3 ratios. In two series of polymerizations carried out for low (samples 9–11) and high (samples 12–14) initial monomer concentration further decreasing of polymers unsaturation was observed. The lowest level occurs at* t*-BuOK*/t*-BuOH/18C6 (1/3/3) (sample 11). The effect of the ligand added was presented in Scheme 6.

In our opinion, nucleophilicity and basicity of crowned ion pair 1′ is greater than that of** 6**. However, in the system activated by the ligand the acceleration of chain transfer to monomer is lower than the acceleration of propagation. Thus, the influence of 18C6 on behavior of ion pairs** 1** is opposite to that observed for hydrogen-bonded ion pairs** 6**. In the first case unsaturation of polymers increases, whereas in the second one it decreases. It presumably results from great acceleration of alcohol deprotonation in the presence of ligand. Distinct decrease of PPO unsaturation was also observed previously in the polymerization initiated* n*-BuOK/*n*-BuOH (1/3) [10] or potassium 1-methoxy-2-propoxide/1-methoxy-2-propanol (1/3) [12] both activated 18C6. However, the authors did not explain this phenomenon. Molar mass of PPOs obtained at 110°C in the presence of* n*-BuOK/*n*-BuOH (1/3) increased gradually from *M*_*n*_ = 1240 without 18C6 and *M*_*n*_ = 2020 for* n*-BuOK/*n*-BuOH/18C6 (1/3/1) to *M*_*n*_ = 2140 for* n*-BuOK/*n*-BuOH/18C6 (1/3/3). In the second system, that is, potassium 1-methoxy-2-propoxide/1-methoxy-2-propanol (1/3), addition of 18C6 for (1/3/0.75 ÷ 4.54) decreases *M*_*n*_ from 3500 to 3300 at 80°C. However, at 40°C for (1/3/1.47 ÷ 1.94) *M*_*n*_ increases from 4050 to 12 400. The authors did not comment on these results. Unexpectedly, ligand 18C6 practically does not influence molar mass of polymers prepared in our work, contrary to the previous data [10, 12]. *M*_*n*_ of polymers 6 and 11 are the same and *M*_*n*_ of polymer 14 is only small higher than that of polymer 8. In general, these effects are probably consistent with great acceleration of chain transfer reaction to* t*-butanol in comparison to the monomer in the presence of 18C6. Further addition of alcohol (samples 15 and 16 in* Set D*) results in decreasing of unsaturation and *M*_*n*_ of polymers.

**Scheme 7:**
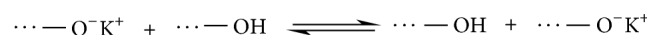
Cation exchange reaction between polymer chains.

Dispersity (*M*_*w*_/*M*_*n*_) of all synthesized polymers in this work is generally low, probably due to high rate of counterion exchange reaction (Scheme 7).

It is also worth noting that the presence of nonpolar solvent, that is,* n*-pentane, in the reaction mixture results in relatively small decrease of polymer unsaturation. For example, unsaturation of PPOs prepared similarly to sample 1, but in mixture of THF/*n*-pentane = 1/1 or 1/5 volume ratios, reaches the values equal to 13.1 or 11.4%, respectively. *M*_*n*_ of polymers is practically unchanged.

Summarizing, anionic polymerization of propylene oxide initiated potassium alkoxide is very sensitive on additives such as counterion complexing ligand and/or hydroxylic compound, that is, alcohol. In general, they strongly influence unsaturation and molar mass of the synthesized polyethers.

## 4. Conclusions

Potassium* t*-butoxide dissolved in tetrahydrofuran is effective initiator of propylene oxide polymerization at room temperature. Polymerizations were carried out at [PO]_o_ and [*t*-BuOK]_o_ equal to 2.0 and 0.1 mol/dm^3^ or 10.0 and 0.05 mol/dm^3^, respectively. The polymers obtained have relatively high unsaturation which changes strongly in the presence of some additives. The main characteristic features of the studied processes are as follows:

(i) In the presence of 18-crown-6 unsaturation of poly(propylene oxide) markedly increases and *M*_*n*_ of polymer decreases due to distinct acceleration of chain transfer reaction to the monomer.
(ii) In this system direct deprotonation of the monomer occurs exclusively and not deprotonation by elimination due to steric hindrance of cation complexed ligand.
(iii) In the presence of* t*-butanol decreasing of unsaturation and *M*_*n*_ of polymers occurs which results from chain transfer reaction to alcohol.
(iv) Activation of the system containing alcohol by 18-crown-6 results in further decrease of polymer unsaturation; however, *M*_*n*_ of polymers (~4800) does not change probably due to great acceleration of chain transfer reaction to alcohol.
(v) This result differs strongly from that reported earlier for PO polymerization initiated potassium 1-methoxy-2-propoxide/1-methoxy-2-propanol in which in the presence of the same ligand *M*_*n*_ increases to ~12 400 for the same ratio of reagents.
(vi) Addition of nonpolar solvent* n*-pentane to the system containing* t*-BuOK results only in small decrease of polymers unsaturation.
(vii) Dispersities of synthesized polymers are relatively low and independent on additives used.
(viii) In general, the observed effects result from different influence of applied additives, as crown ether and/or alcohol on the rates of propagation and chain transfer to the monomer and alcohol as well as cation exchange reactions.

## Figures and Tables

**Table 1:** Characterization of unsaturation of PPOs synthesized in the presence of *t*-BuOK including 18C6 or *t*-BuOH or *t*-BuOH/18C6 as additives.

Sample	Initiating system	Macromolecules with unsaturated starting groups (mol %)	*M*_*n*_ (SEC)	*M*_*w*_/*M*_*n*_ (SEC)
*Set A*				
1	*t*-BuOK	14.0	2600	1.05
2	*t-*BuOK/18C6 (1/1)	26.8	2300	1.05
3	*t*-BuOK	28.4	6400	1.07
4	*t-*BuOK/18C6 (1/1)	40.1	5800	1.06
*Set B*				
5	*t-*BuOK/*t*-BuOH (1/1)	10.1	1700	1.07
6	*t-*BuOK/*t*-BuOH (1/3)	1.3	1000	1.10
7	*t-*BuOK/*t*-BuOH (1/1)	22.5	6000	1.08
8	*t-*BuOK/*t*-BuOH (1/3)	12.0	4700	1.12
*Set C*				
9	*t-*BuOK/*t*-BuOH/18C6 (1/3/1)	0.5	900	1.06
10	*t-*BuOK/*t*-BuOH/18C6 (1/3/2)	0.3	1000	1.04
11	*t-*BuOK/*t*-BuOH/18C6 (1/3/3)	0.2	1000	1.05
12	*t-*BuOK/*t*-BuOH/18C6 (1/3/1)	4.1	4800	1.02
13	*t-*BuOK/*t*-BuOH/18C6 (1/3/2)	3.5	4800	1.09
14	*t-*BuOK/*t*-BuOH/18C6 (1/3/3)	3.3	4900	1.04
*Set D*				
15	*t-*BuOK/*t*-BuOH/18C6 (1/5/3)	1.1	4100	1.03
16	*t-*BuOK/*t*-BuOH/18C6 (1/7/3)	0.3	3700	1.04

## References

[1] Gosa K.-L., Uricanu V. (2002). Emulsions stabilized with PEO-PPO-PEO block copolymers and silica. *Colloids and Surfaces A: Physicochemical and Engineering Aspects*.

[2] Zhang Z., Xu G. Y., Wang F., Dong S. I., Li Y. M. (2004). Characterization and demulsification of poly(ethylene oxide)–block–poly(propylene oxide)–block–poly(ethylene oxide) copolymers. *Journal of Colloid and Interface Science*.

[3] de Lucas A., Rodríguez L., Pérez-Collado M., Sánchez P. (2002). Production of polyether polyols using caesium as catalyst. *Polymer International*.

[4] Mathur A. M., Drescher B., Scranton A. B., Klier J. (1998). Polymeric emulsifiers based on reversible formation of hydrophobic units. *Nature*.

[5] Jeong B., Bae Y. H., Lee D. S., Kim S. W. (1997). Biodegradable block copolymers as injectable drug-delivery systems. *Nature*.

[6] Cendejas G., Arreguín F., Flores C., Villalobos I., Flores E., Vázquez F. (2008). Novel initiators for the synthesis of propylene oxide oligomers by anionic ring opening polymerization. *Catalysis Today*.

[7] Wegener G., Brandt M., Duda L. (2001). Trends in industrial catalysis in the polyurethane industry. *Applied Catalysis A: General*.

[8] Ionescu M. (2005). *Chemistry and Technology of Polyols for Polyurethanes*.

[9] Becker H., Wagner G., Stolarzewicz A. (1982). Zur Übertragungsreaktion bei der anionischen Polymerisation von Oxiranen. III. Zur Dynamik der Doppelbindungsbildung bei der Propylenoxidpolymerisation. *Acta Polymerica*.

[10] Becker H., Wagner G. (1984). Zur Übertragungsreaktion bei der anionichen Polymerisation von Oxiranen VI. Zum Einfluß von Kronenetherzusätzen auf die Polymerisation von Propylenoxid. *Acta Polymerica*.

[11] Ionescu M., Mihalache I., Dumitriu V., Stoenescu F., Ion V. (1988). Anionic-Polymerization of the propylene oxide with the complexed alkali opposed ion. *Revista de Chimie*.

[12] Ding J., Heatley F., Price C., Booth C. (1991). Use of crown ether in the anionic polymerization of propylene oxide—2. Molecular weight and molecular weight distribution. *European Polymer Journal*.

[13] Siggia S. (1963). *Quantitative Organic Analysis via Functional Groups*.

[14] Grobelny Z., Matlengiewicz M., Jurek J. (2013). The influence of macrocyclic ligands and water on propylene oxide polymerization initiated with anhydrous potassium hydroxide in tetrahydrofuran. *European Polymer Journal*.

